# Camouflage Orthodontic Treatment of a Severe Class III Malocclusion

**DOI:** 10.1155/crid/9839448

**Published:** 2025-01-04

**Authors:** Mandla Dominic Nyakale

**Affiliations:** ^1^ Department of Orthodontics, School of Dentistry, Sefako Makgatho Health Sciences University, Pretoria, South Africa, smu.ac.za

**Keywords:** camouflage orthodontic treatment, Class III elastics, Class III malocclusion, dental compensation

## Abstract

Class III malocclusion remains the most challenging occlusal problem to treat due to the complexity of the interrelationships of the underlying skeletal and dental structures. Camouflage orthodontic treatment is a preferred alternative method used to manage mild to moderate Class III malocclusion in nongrowing patients. The aim of this article was to demonstrate a camouflage orthodontic treatment of a 22‐year‐old female patient diagnosed as having a severe skeletal Class III malocclusion characterized by a straight facial profile, reverse overjet, crowded maxillary incisors, retrognathic maxilla, prognathic mandible, and a hypodivergent facial pattern. The initial diagnosis suggested orthognathic surgery, but the patient preferred the alternative treatment. The treatment approach chosen was nonextraction camouflage orthodontic treatment combined with Class III elastics. At the end of treatment, the canines and molars were in a Class I relationship, while the incisors presented a normal overjet and overbite relationship. The maxillary and mandibular midlines corresponded with each other and with the midsagittal plane, and the smile aesthetics were improved. The facial profile was slightly improved, and the vertical face height was also slightly increased with treatment. The treatment results were maintained 15 months after treatment. It was concluded that camouflage orthodontic treatment can be used as an effective alternative method to achieve acceptable dentofacial aesthetics, functional occlusion, and stability in treating an adult patient diagnosed with severe skeletal Class III malocclusion characterized by a retrognathic maxilla, prognathic mandible, and a hypodivergent facial pattern.

## 1. Introduction

Class III malocclusion is one of the most challenging occlusal problems to treat. It is characterized by a variety of dental and skeletal features which may vary from what is ideal occlusion. The clinical features of Class III malocclusion include a retrognathic maxilla and/or a prognathic mandible [1] with dentoalveolar compensations [2]. According to Zere et al. [3], the most frequent pattern of Class III malocclusion is the combination of a retrognathic maxilla with a normal mandible. The evolution of Class III malocclusion can further be complicated by the presence of other vertical and transverse skeletal discrepancies [4–9], and this is largely because growth cannot be predicted with accuracy. Many patients with Class III malocclusion often present with compromised facial aesthetics, and this is one of the reasons why they seek treatment in the first place [10]. The prevalence of Class III malocclusion is low in the general population; however, the highest prevalence was recorded in the Chinese population and the lowest prevalence in the Indian population [11]. In South Africa, Jacobson [12] reported a prevalence of 0.7%. Class III malocclusion has a multifactorial etiology which includes genetic [13], developmental [14], and environmental factors [15]. Clinical examination, functional, cephalometric, and study model analyses are typically used to make a diagnosis.

Class III malocclusion is one of the most challenging occlusal problems to treat due to the complex nature of the interactions between the underlying dentoskeletal structures [16–18]. The choice of treatment for Class III malocclusion usually depends on the severity of malocclusion and the degree of skeletal maturity of the patient. In growing patients, treatment can be achieved with growth modification appliances such as protraction face mask appliances [19]. In nongrowing adult patients, treatment is more complex due to the limited number of options available [20]. In severe cases, orthodontic treatment combined with orthognathic surgery is usually the ideal treatment [21]; however, many patients often refuse this treatment because of its high cost and the invasive nature of surgery [22]. According to Troy et al. [22], nongrowing adult patients with mild to moderate skeletal Class III malocclusion and acceptable facial aesthetics can benefit from camouflage orthodontic treatment [23]. Camouflage orthodontic treatment usually involves the proclination of maxillary incisors and retroclination of mandibular incisors to establish normal overjet and overbite relationships of the incisors without changing the underlying skeletal discrepancy [24]. The aim of this article was to discuss camouflage orthodontic treatment as an alternative in treating an adult patient diagnosed with a severe Class III malocclusion characterized by a retrognathic maxilla, prognathic mandible, and hypodivergent facial pattern.

## 2. Case Presentation

**Figure 1:**
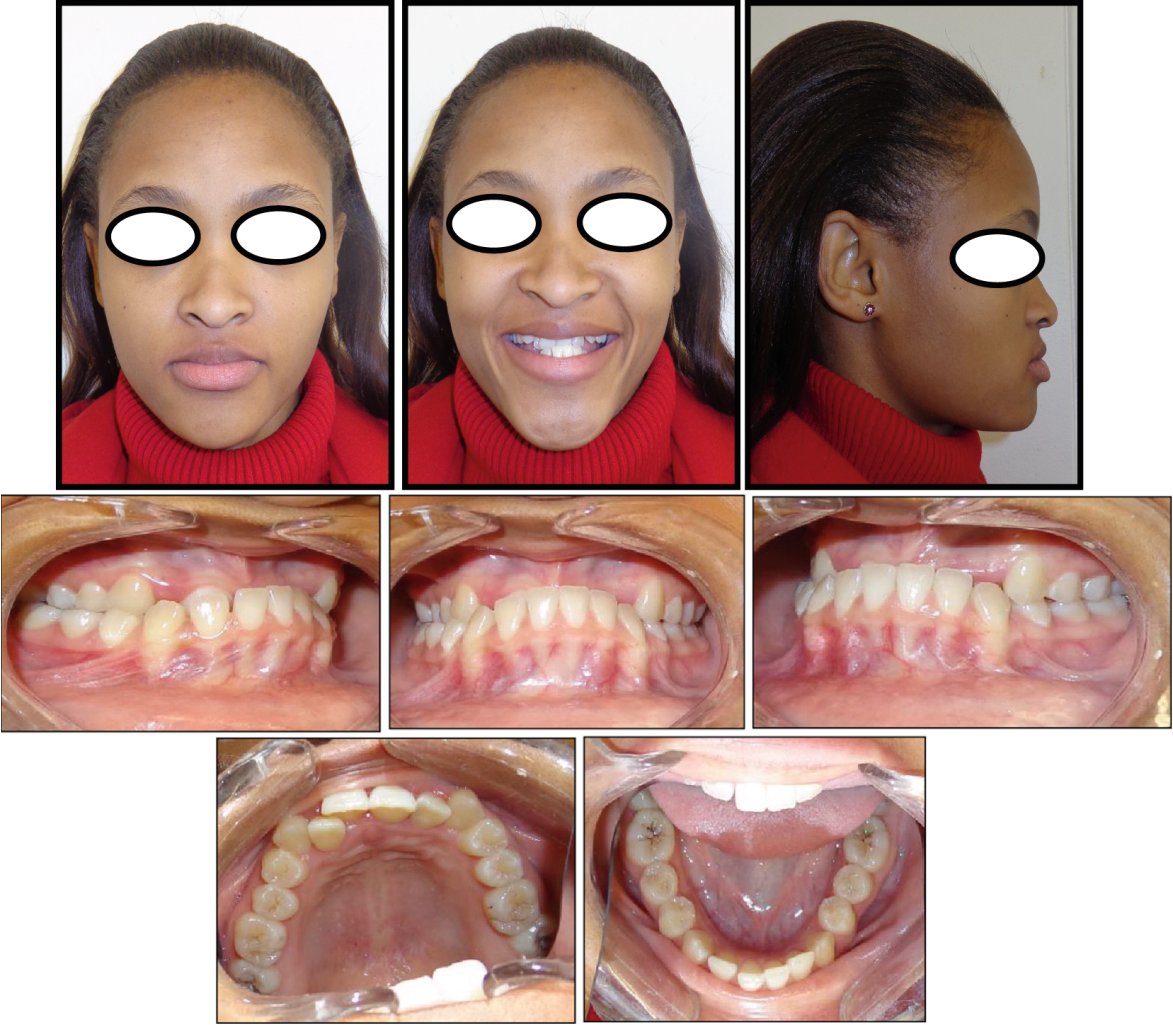
Pretreatment facial and intraoral photographs.

A 22‐year‐old female patient was referred to the orthodontic clinic at Pelonomi Tertiary Hospital in Bloemfontein (Free State Province, South Africa) with the chief complaint of crowded maxillary anterior teeth that bite behind the mandibular teeth and poor aesthetics. Clinical examination was systematically conducted in accordance with the method described by the author in the previous study [25]. The patient was in good general health, and she had no medical condition which could prevent her from receiving orthodontic treatment. The patient had no history of trauma suffered to the head and neck area, and no signs or symptoms of temporomandibular joint dysfunction were noted at the time of initial examination. Extraoral facial analysis showed a hypodivergent facial pattern and a mesoprosopic facial form with a symmetrical face (Figure 1). Analysis of the face in the sagittal plane showed a straight facial profile, with competent lips. Intraoral soft tissue examination showed healthy periodontal and gingival tissues with no evidence of bleeding or deep probing depths. The patient was in the permanent dentition stage, and the teeth were generally healthy with no carious lesions (Figure 1). There was a significant amount of crowding in the maxillary arch and minimal crowding in the mandibular arch. The molars were in Class III relationship on both the left and right sides, and the canines were in Class III relationship on the right side and Class I on the left side. The overjet was measured as negative 5 mm, and the overbite was measured as 12 mm with an accentuated curve of Spee. The molars and premolars on the right were in a crossbite relationship. The maxillary midline was deviated 3 mm to the right, and the mandibular midline was coincident with the midsagittal plane. The temporomandibular joint was asymptomatic both at rest and during mandibular movements. The patient also reported no history of Class III malocclusion in her immediate family and among her close relatives.

**Figure 2:**
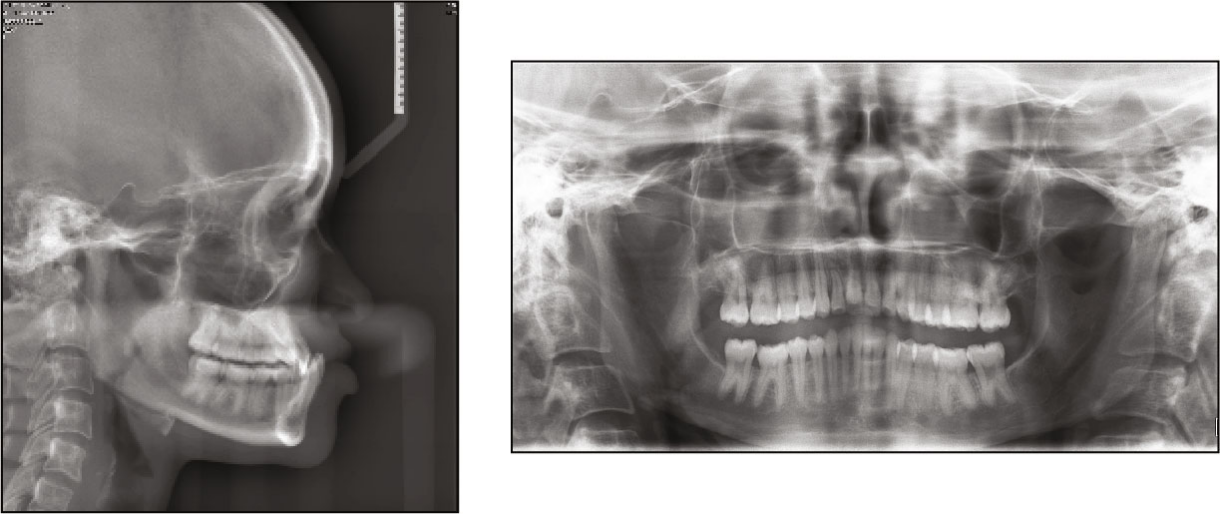
Pretreatment radiographs.

Analysis of the panoramic radiograph showed the teeth in the permanent dentition stage with generalized shortening of the roots, especially the maxillary incisors and mandibular molars (Figure 2). The third molars were missing both in the maxilla and mandible, and no other pathologies were detected. Analysis of a lateral cephalometric radiograph showed a severe Class III sagittal jaw relationship with a retrognathic maxilla and a prognathic mandible (Figure 2 or Table 1). Analysis of the growth pattern showed a hypodivergent facial pattern, and other cephalometric measurements are summarized in Table 1. Periapical radiographs were not available for evaluation.

**Table 1:** Cephalometric measurements.

**Measurement**	**Normal value**	**Pretreatment**	**Posttreatment**
Skeletal pattern			
SNA (°)	87°	82°	82°
SNB (°)	82°	93°	86°
ANB (°)	5°	−11°	−4°
Face plane (°)	88–90°	100°	94°
Convexity (mm)	4 mm	−9 mm	−4 mm
Wits (mm)	−1 to 2 mm	−11 mm	−5 mm
Growth pattern			
SN‐FH (°)	7°	8°	10°
SN‐Occl (°)	16°	9°	14°
SN‐MP (°)	32–34°	15°	22°
*y*‐axis (°)	66–68°	59°	65°
UFH:LFH	5:7	5:6	5:7
Incisor relations			
U1‐NA (°)	22°	28°	30°
U1‐NA (mm)	7 mm	11 mm	13 mm
L1‐NB (°)	38°	17°	23°
L1‐NB (mm)	10 mm	4 mm	7 mm
APo (mm)	8 mm	7 mm	6 mm
Soft tissue relations			
Holdaway (°)	20°	10°	15°

## 3. Treatment Objectives

The objectives of treatment were to create the space required to level and align the teeth, correct dental midlines, correct the anterior and posterior crossbites, flatten out the curve of Spee, establish good static and functional occlusions, and improve facial aesthetics. Three possible treatment options were discussed with the patient before the final decision was made. The first treatment option was a combination of orthodontics and surgical treatment with a high LeFort 1 osteotomy to advance the maxilla and mandibular osteotomy to set back the mandible to improve facial appearance. The second treatment option consisted of extraction of the mandibular first premolars and retraction of the lower canines and incisors to establish a positive overjet. The third treatment option was a nonextraction camouflage orthodontic treatment approach using Class III elastics. After discussing the advantages and disadvantages of each treatment option, the patient chose the nonextraction camouflage orthodontic treatment approach. The inclusion criteria for this treatment approach were a skeletal Class III malocclusion characterized by a retrognathic maxilla, prognathic mandible, and a hypodivergent facial pattern.

## 4. Treatment Progress

**Figure 3:**
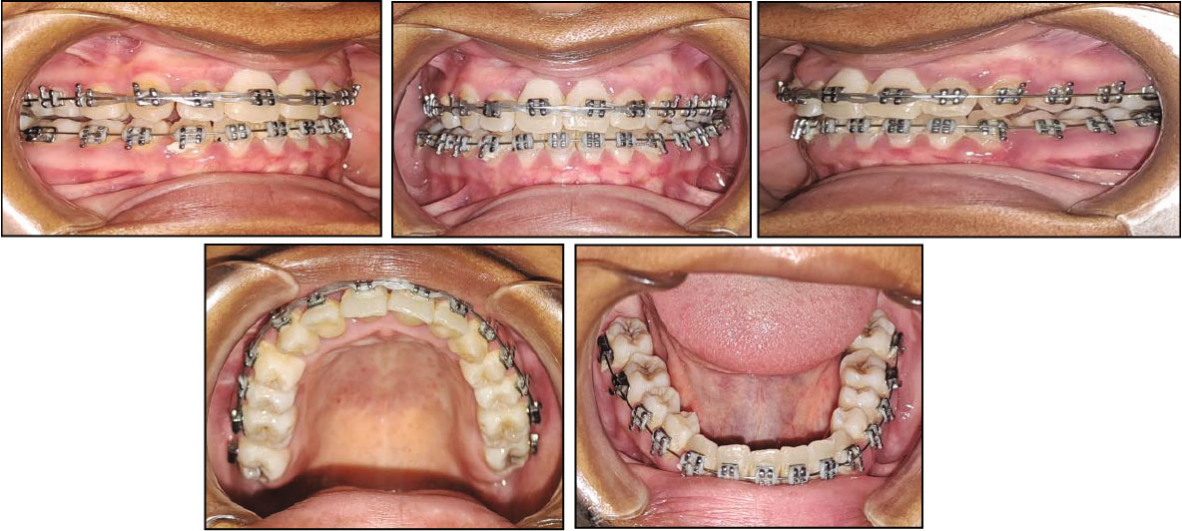
Intermediate intraoral photographs.

Teeth were bonded with the standard preadjusted 0.018 × 0.025‐inch slot Roth prescription edgewise brackets. The bracket on the maxillary right lateral incisor was rotated 180° to increase the labial root torque. Buttons were bonded on the palatal aspects of the right maxillary molars and premolars. An occlusal splint was placed on the occlusal surfaces of the mandibular molars to open the bite to prevent the mandibular incisors from contacting the maxillary incisor brackets. Teeth were levelled and aligned with 0.012‐, 0.014‐, and 0.016‐inch superelastic nickel–titanium archwires, respectively, and at this point, the maxillary right lateral incisor was not included. After the teeth were levelled and aligned, a 0.016‐inch stainless steel archwire was placed in the maxillary arch with a nickel titanium open‐coil spring to open space for the alignment of the maxillary right lateral incisor. Levelling and aligning archwires were placed again to finalize the alignment of all the teeth including the maxillary right lateral incisor. Distal segments of the mandibular archwire were cut off on both the left and right sides, and vertical elastics were placed to facilitate overeruption of the premolars until they were in occlusion with their opposing counterparts (Figure 3). The occlusal splint was then removed, and the vertical elastics were extended more posteriorly to facilitate the overeruption of the molars. Class III elastics were initiated, and the patient was encouraged to wear them until the overjet was corrected. Crossbite elastics were also placed to correct the posterior crossbite on the right buccal segment. Torque control was initiated with 0.016 × 0.022‐inch superelastic nickel titanium, followed by 0.017 × 0.025‐inch beta titanium archwires, respectively. Final detailing of the occlusion was done using Class III elastics on a 0.018 × 0.025‐inch stainless steel archwire in the maxillary arch and a 0.018‐inch stainless steel archwire in the mandibular arch. The final rectangular stainless‐steel archwire was left in place in the maxillary arch for an additional 8 weeks to establish proper labial root torque, especially on the right maxillary lateral incisor. At the end of treatment, the orthodontic appliances were removed, and maxillary and mandibular Hawley retainers were placed.

## 5. Treatment Results

**Figure 4:**
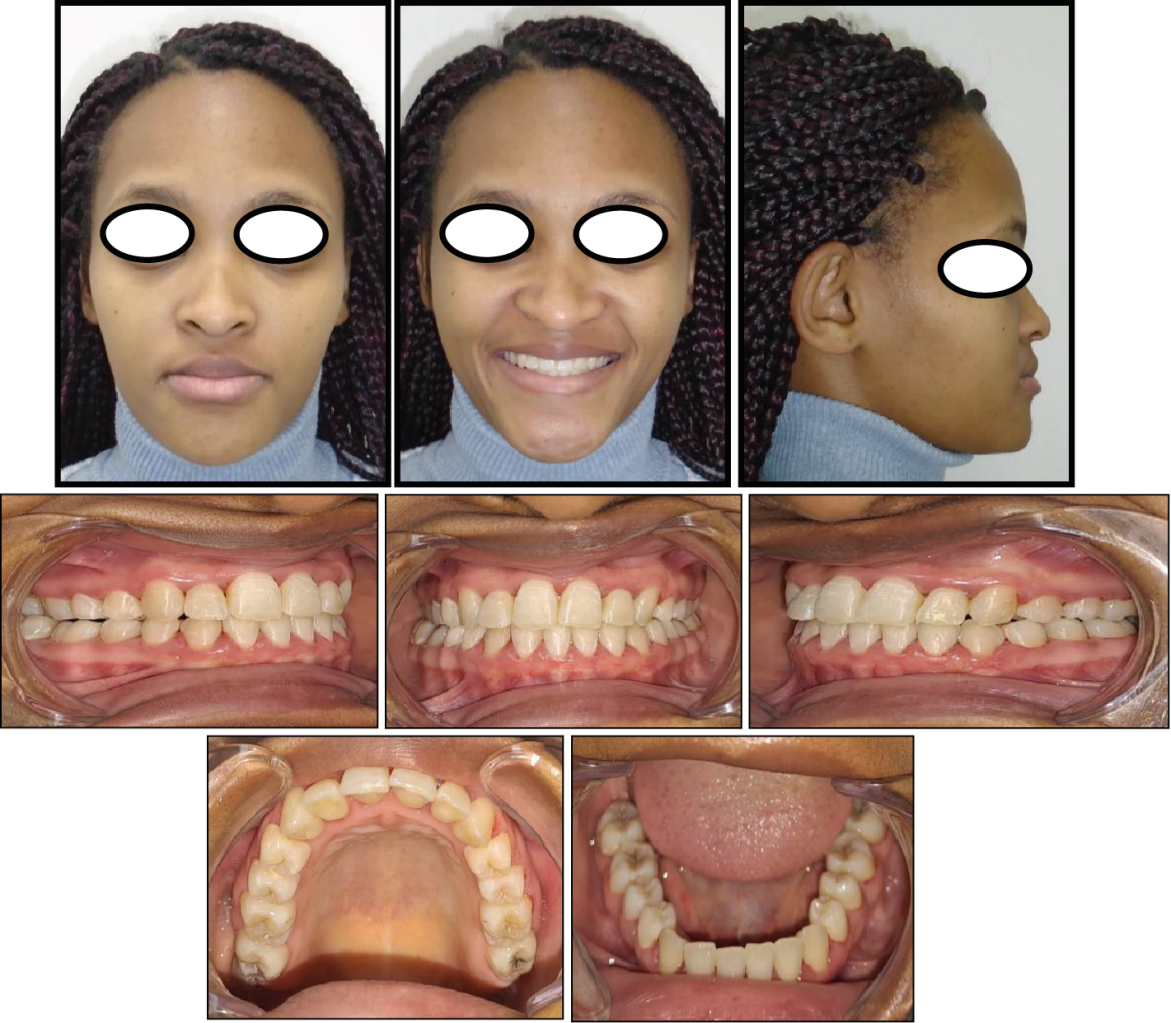
Posttreatment facial and intraoral photographs.

**Figure 5:**
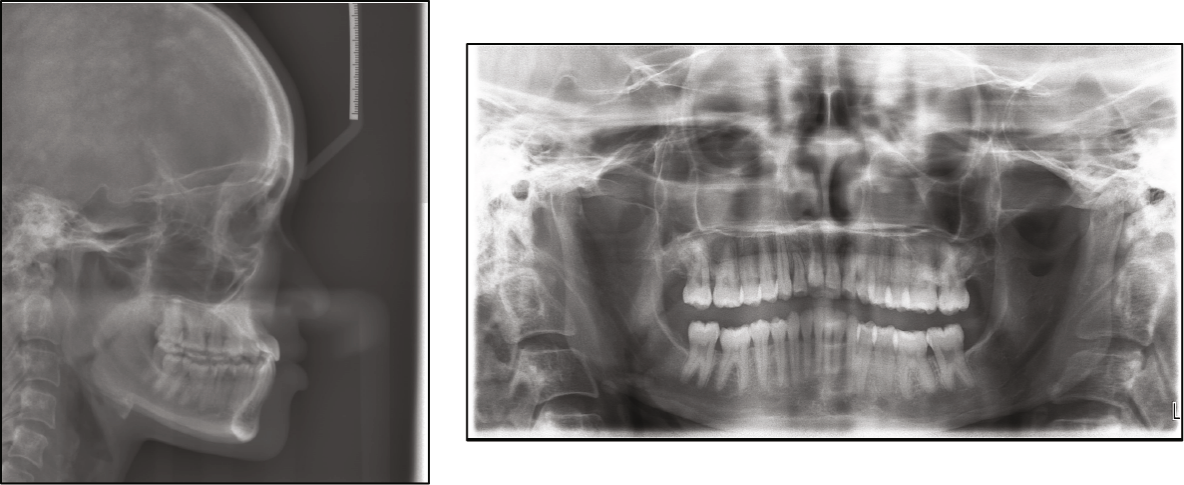
Posttreatment radiographs.

**Figure 6:**
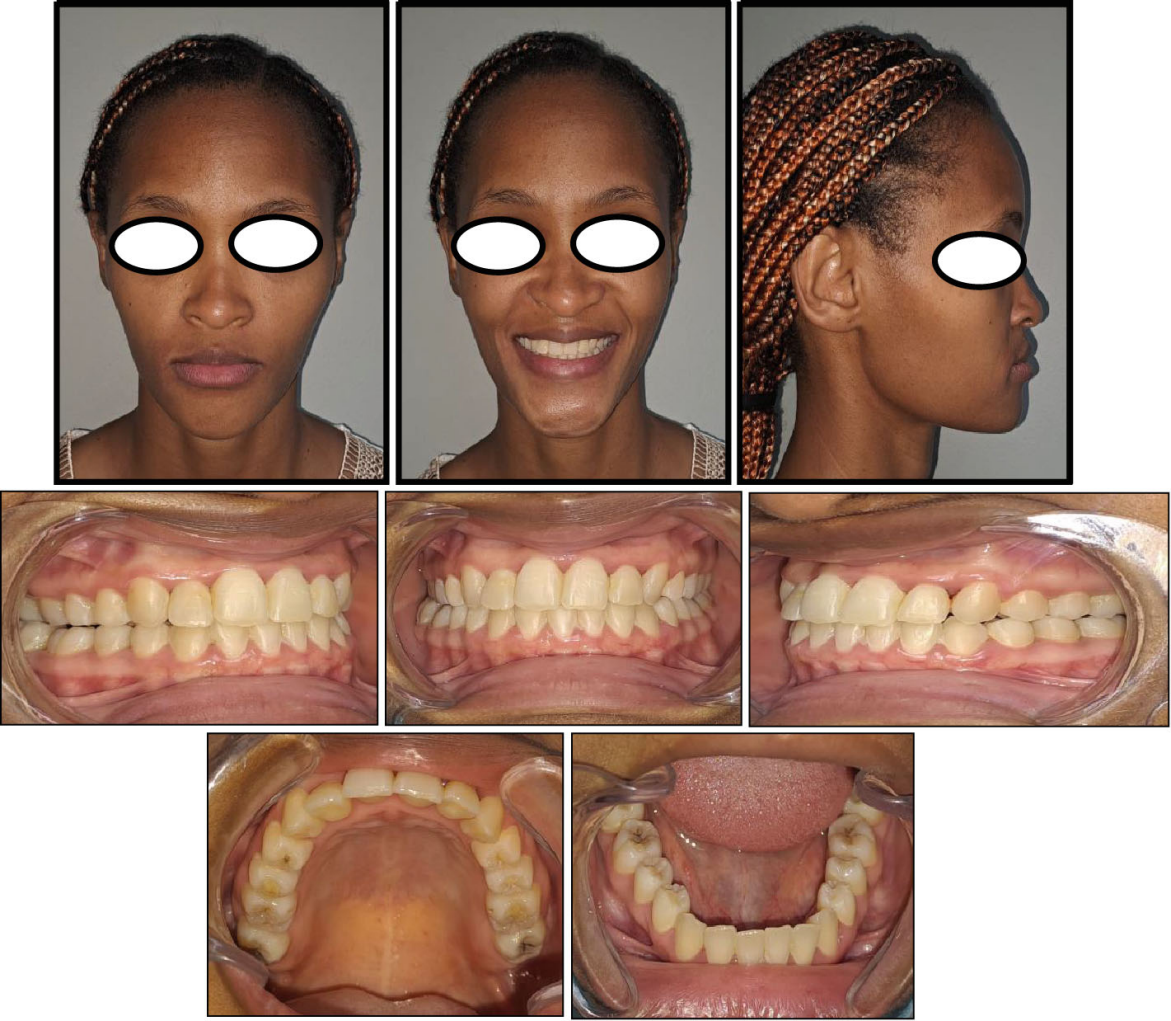
Postretention facial and intraoral photographs.

Treatment lasted for 27 months, and at the end of orthodontic treatment, the teeth were levelled and aligned (Figure 4). The canines and molars were in a Class I relationship, with normal overjet and overbite relationship of the incisors. The maxillary and mandibular midlines corresponded with each other and with the midsagittal plane, which improved the smile aesthetics significantly. A posttreatment panoramic radiograph showed the optimal positioning of the maxillary and mandibular teeth with proper root parallelism (Figure 5). There was also no sign of alveolar bone or root resorption on the panoramic radiograph. Posttreatment lateral cephalometric analysis showed a significant increase in the angle formed by Point A to nasion line and nasion to Point B line (ANB angle) together with all the other vertical measurements, and there was also a slight improvement in the facial profile (Figure 5 or Table 1). Records taken after 15 months of retention showed that the treatment results were maintained during the retention period (Figure 6), and the patient was satisfied with the treatment results. The gingiva was healthy, and the periodontal attachment of the previously displaced maxillary right lateral incisor was still intact.

## 6. Discussion

The present case report is aimed at discussing the orthodontic treatment of an adult patient diagnosed with Class III malocclusion. To the best of our knowledge, such a severe case of Class III malocclusion treated without surgery has never been reported in the literature. The outcome of treatment of this case will give us some insight into the treatment options available to us regarding this malocclusion. The typical clinical features of Class III malocclusion include a concave facial profile due to a retrognathic maxilla and/or a prognathic mandible, and dental compensations are often seen [1]. Our patient presented with a straight facial profile, reverse overjet relationship of the incisors, and a deep bite tendency (Figure 1). This case presented several treatment options that were discussed with the patient, and each option was weighed against its advantages and disadvantages before the final decision was made. Growth modification treatment was not regarded as a treatment option because we expected skeletal growth in our patient to have ceased at her age [26–28]. In most adult patients, orthognathic surgery is often the ideal treatment for Class III malocclusion [29]; however, our patient did not want to undertake this treatment, and this left us with camouflage orthodontic treatment as the only available option. Camouflage orthodontic treatment involves the movement of teeth into the compensatory positions that will improve occlusion without changing the underlying skeletal discrepancy [29].

Several authors have shown that adult patients with mild to moderate skeletal Class III malocclusion and acceptable facial aesthetics can be treated successfully with camouflage orthodontic treatment [20, 23, 24]; however, Burns et al. [24] stated that camouflage orthodontic treatment also has its limitations. Before beginning any treatment, we must carefully analyze three clinical criteria that can be used to predict therapeutic success or failure. First, we need to assess the extent of compromise of the facial aesthetics and how important this is for the patient’s psychosocial well‐being [30, 31]. In case of significant aesthetic concern, Bell et al. [28] recommend that orthognathic surgery should be undertaken to improve the patient’s profile and ultimately their psychosocial well‐being. Our patient had a straight facial profile, and this did not negatively affect her psychosocial well‐being. Secondly, we need to assess the position and inclination of the maxillary and mandibular incisors to determine whether it will be possible to move them into the compensatory positions that will achieve good occlusion with improved dental aesthetics [2, 24, 32, 33]. In the present case, the maxillary incisors were proclined and protruded, and the mandibular incisors were retroclined and retruded (Figure 2 or Table 1); this degree of dentoalveolar compensation usually warrants orthognathic surgery, but the patient wanted to avoid that. The maxillary arch crowding was favourable for advancing the incisors, and in addition, the deep bite and an accentuated curve of Spee were favourable for molar extrusion and clockwise rotation of the mandible. Thirdly, we need to assess the thickness of the mandibular symphysis to determine whether it will allow significant retraction of the mandibular incisors [34–36]. Our patient had a narrow mandibular symphysis (Figure 2), which was not favourable for the retraction of the incisors. Garib et al. [35] stated that retraction movements of mandibular incisors in Class III subjects with narrow mandibular symphyses should not be undertaken to avoid the risk of damage to periodontal tissue and also to minimize the relapse tendency. It is for this reason that we wanted to avoid extractions in the mandibular arch, but instead, we relied on the use of Class III elastics.

At the end of treatment, we also noticed a significant increase in the ANB angle (Figure 4 or Table 1), and this improved the underlying Class III skeletal discrepancy. Opening the bite using the occlusal splints caused the mandible to rotate in a clockwise direction, and according to Jacobson [36], clockwise rotation of the mandible will result in distal positioning of Point B and subsequent increase in the ANB angle. In addition, Hussels and Nanda [37] pointed out that an increase in the vertical dimension (as indicated by an increase in the distance between nasion and Point B), as well as an increase in the dental height (as indicated by an increase in the distance between Points A and B), may contribute to an increase in ANB angle. The other indicators of improved anteroposterior and vertical dentoskeletal features were shown by the increase in occlusal and sella‐nasion (SN‐Occl) plane angle, sella‐nasion to mandibular plane (SN‐MP) angle, *y*‐axis angles, and lower anterior face height; these are common effects of Class III orthodontic mechanics [38–40]. The results of this case have demonstrated that Class III malocclusion, although complicated, can be treated successfully with meticulous treatment planning and applying orthodontic mechanics appropriately; however, patient selection is of paramount importance in achieving the best possible results [24]. Although the results of this case were satisfactory, this case also presented with two disadvantages. The first disadvantage was poor labial root torque control on the maxillary right lateral incisor. Adequate torque control on the teeth can be achieved by applying a large moment‐to‐force ratio of at least 12:1 [41], and this can be achieved by placing a full‐thickness stainless steel archwire for a prolonged period of time. Unfortunately, the patient desired to discontinue treatment because she felt that her treatment had gone on for too long and wanted to stop. The second disadvantage was our inability to properly assess the presence and extent of external apical root resorption. Analysis of the panoramic radiograph showed no signs of external apical root resorption, especially in relation to the maxillary and mandibular incisors (Figure 4). A study by Sameshima and Asgarifar [41] has shown that periapical radiographs are more accurate when evaluating the presence and extent of external apical root resorption, but unfortunately, these radiographs were not available in the hospital when the initial and posttreatment records were taken. Despite the challenges this case presented, we were able to produce reasonably acceptable dentofacial aesthetics and functional occlusion. More importantly, though, the patient was pleased with the treatment results.

## 7. Conclusion

It was concluded that camouflage orthodontic treatment can be used as effective alternative method to achieve acceptable dentofacial aesthetics, functional occlusion, and stability in treating an adult patient diagnosed with severe skeletal Class III malocclusion characterized by a retrognathic maxilla, prognathic mandible, and a hypodivergent facial pattern.

## Consent

Permission to use the hospital records was sought from the head of the Department of Orthodontics, the chief executive officer of Pelonomi Hospital, and the head of the Department of Health of the Free State Province. The patient has granted permission for the use of her pictures and other pertinent clinical data in this article solely for educational and research purposes. A copy of the written informed consent is available for review by the editor‐in‐chief of this journal.

## Conflicts of Interest

The author declares no conflicts of interest.

## Funding

The author declares that this article is self‐funded and is not supported by any external sources of funding.

## Data Availability

The data that support the findings of this study are available on request from the corresponding author. The data are not publicly available due to privacy or ethical restrictions.

## References

[1] Ngan P. and Moon W. , Evolution of Class III treatment in orthodontics, American Journal of Orthodontics and Dentofacial Orthopedics. (2015) 148, no. 1, 22–36, 10.1016/j.ajodo.2015.04.012, 2-s2.0-84940976757, 26124025. 26124025

[2] Janson G. , de Souza J. E. , de Andrade Alves F. , Andrade P.Jr., Nakamura A. , de Freitas M. R. , and Henriques J. F. , Extreme dentoalveolar compensation in the treatment of Class III malocclusion, American Journal of Orthodontics and Dentofacial Orthopedics. (2005) 128, no. 6, 787–794, 10.1016/j.ajodo.2004.08.018, 2-s2.0-29144534266. 16360922

[3] Zere E. , Chaudhari P. K. , Sharan J. , Dhingra K. , and Tiwari N. , Developing Class III malocclusions: challenges and solutions, Clinical, Cosmetic and Investigational Dentistry. (2018) 10, 99–116, 10.2147/CCIDE.S134303, 2-s2.0-85049579968, 29950903. 29950903 PMC6016584

[4] Yang C. and Tseng Y. , The orthodontic treatment of Class III malocclusion with anterior crossbite and severe deep bite, Taiwanese Journal of Orthodontics. (2019) 31, no. 1, 53–63.

[5] Staudt C. B. and Kiliaridis S. , Different skeletal types underlying Class III malocclusion in a random population, American Journal of Orthodontics and Dentofacial Orthopedics. (2009) 136, no. 5, 715–721, 10.1016/j.ajodo.2007.10.061, 2-s2.0-71849089268, 19892290. 19892290

[6] Ellis E. E. and McNamara J. A. , Components of adult Class III malocclusion, Journal of Oral and Maxillofacial Surgery. (1984) 42, no. 5, 295–305, 10.1016/0278-2391(84)90109-5, 2-s2.0-0021327420. 6585502

[7] Guyer E. C. , Ellis E. E. , McNamara J. A. , and Behrents R. G. , Components of Class III malocclusion in juveniles and adolescents, Angle Orthodontist. (1986) 56, no. 1, 7–30, 3485393. 3485393 10.1043/0003-3219(1986)056<0007:COCIMI>2.0.CO;2

[8] Jacobson A. , Evans W. G. , Preston C. B. , and Sadowsky P. L. , Mandibular prognathism, American Journal of Orthodontics. (1974) 66, no. 2, 140–171, 10.1016/0002-9416(74)90233-4, 2-s2.0-0016155278. 4526387

[9] Williams S. and Andersen C. E. , The morphology of the potential Class III skeletal pattern in the growing child, American Journal of Orthodontics. (1986) 89, no. 4, 302–311, 10.1016/0002-9416(86)90052-7, 2-s2.0-0022445939, 3457529. 3457529

[10] Motloba D. P. , Sethusa M. P. S. , and Ayo-Yusuf A. O. , The psychological impact of malocclusion on patients seeking orthodontic treatment at a South African oral health training centre, South African Dental Journal. (2002) 71, 200–205.

[11] Hardy D. K. , Cubas Y. P. , and Orellana M. F. , Prevalence of angle Class III malocclusion: a systematic review and meta-analysis, Open Journal of Epidemiology. (2012) 2, no. 4, 75–82, 10.4236/ojepi.2012.24012.

[12] Jacobson A. , Occlusion and malocclusion in the South African Bantu, The Journal of the Dental Association of South Africa. (1967) 67, 125–138. 5248125

[13] Harris E. F. and Johnson M. G. , Heritability of craniometric and occlusal variables: a longitudinal sib analysis, American Journal of Orthodontics and Dentofacial Orthopedics. (1991) 99, no. 3, 258–268, 10.1016/0889-5406(91)70007-J, 2-s2.0-0026133887, 1998301. 1998301

[14] Proffit W. R. , On the aetiology of malocclusion, British Journal of Orthodontics. (1986) 13, no. 1, 1–11, 10.1179/bjo.13.1.1, 2-s2.0-0022567107. 3510662

[15] Todor B. I. , Scrobota I. , Todor L. , Lucan A. I. , and Vaida L. L. , Environmental factors associated with malocclusion in children population from mining areas, western Romania, International Journal of Environmental Research and Public Health. (2019) 16, no. 18, 3383–3390, 10.3390/ijerph16183383, 2-s2.0-85072582436, 31547435. 31547435 PMC6765924

[16] Mackay F. , Jones J. A. , Thompson R. , and Simpson W. , Craniofacial form in Class III cases, British Journal of Orthodontics. (1992) 19, no. 1, 15–20, 10.1179/bjo.19.1.15, 2-s2.0-0026810257, 1562574. 1562574

[17] Battagel J. M. , The aetiological factors in Class III malocclusion, European Journal of Orthodontics. (1993) 15, no. 5, 347–370, 10.1093/ejo/15.5.347, 2-s2.0-0027685732. 8223970

[18] Vaughn G. A. , Mason B. , Moon H. B. , and Turley P. K. , The effects of maxillary protraction therapy with or without rapid palatal expansion: a prospective, randomized clinical trial, American Journal of Orthodontics and Dentofacial Orthopedics. (2005) 128, no. 3, 299–309, 10.1016/j.ajodo.2005.04.030, 2-s2.0-24944590453. 16168327

[19] Park J. H. , Emamy M. , and Lee S. H. , Adult skeletal Class III correction with camouflage orthodontic treatment, American Journal of Orthodontics and Dentofacial Orthopedics. (2019) 156, no. 6, 858–869, 10.1016/j.ajodo.2018.07.029, 31784020. 31784020

[20] Costa P. T. M. , Torrent U. J. M. , and Correia P. J. G. , Orthodontic camouflage in the case of a skeletal Class III malocclusion, World Journal of Orthodontics. (2004) 5, 213–223. 15612340

[21] Jawaid M. , Qadeer T. A. , and Fahim M. F. , Reasons for refusing orthognathic surgery by orthodontic patients: a cross-sectional survey, JPMA. The Journal of the Pakistan Medical Association. (2022) 72, no. 10, 1954–1962, 10.47391/JPMA.3154, 36660981. 36660981

[22] Troy B. A. , Shanker S. , Fields H. W. , Vig K. , and Johnston W. , Comparison of incisor inclination in patients with Class III malocclusion treated with orthognathic surgery or orthodontic camouflage, American Journal of Orthodontics and Dentofacial Orthopedics. (2009) 135, no. 2, 146.e1–146.e9, 10.1016/j.ajodo.2008.07.012, 2-s2.0-59349103283. 19201319

[23] Burns N. R. , Musich D. R. , Martin C. , Razmus T. , Gunel E. , and Ngan P. , Class III camouflage treatment: what are the limits?, American Journal of Orthodontics and Dentofacial Orthopedics. (2010) 137, no. 1, 9.e1–9.e13, 10.1016/j.ajodo.2009.05.017, 2-s2.0-72649088889. 20122418

[24] Nyakale M. D. , Orthodontic treatment of bilateral transposition of maxillary canines and lateral incisors, Case Reports in Dentistry. (2022) 2022, 9, 10.1155/2022/8094008, 35028162, 8094008. 35028162 PMC8752299

[25] Foley T. F. and Mamandras A. H. , Facial growth in females 14 to 20 years of age, American Journal of Orthodontics and Dentofacial Orthopedics. (1992) 101, no. 3, 248–254, 10.1016/0889-5406(92)70094-Q, 2-s2.0-0026831191. 1539552

[26] DiBiase A. T. , Seehra J. , Papageorgiou S. N. , and Cobourne M. T. , Do we get better outcomes from early treatment of Class III discrepancies?, British Dental Journal. (2022) 233, no. 3, 197–201, 10.1038/s41415-022-4507-0, 35962090. 35962090 PMC9374590

[27] Sarangal H. , Namdev R. , Garg S. , Saini N. , and Singhal P. , Treatment modalities for early management of class III skeletal malocclusion: a case series, Contemporary Clinical Dentistry. (2020) 11, no. 1, 91–96, 10.4103/ccd.ccd_393_19. 33110317 PMC7580742

[28] Bell R. , Kiyak H. A. , Joondeph D. R. , McNeill R. W. , and Wallen T. R. , Perceptions of facial profile and their influence on the decision to undergo orthognathic surgery, American Journal of Orthodontics. (1985) 88, no. 4, 323–332, 10.1016/0002-9416(85)90132-0, 2-s2.0-0022375940. 3863490

[29] Bou Wadi M. N. , Freitas K. M. , Freitas D. S. , Cançado R. H. , Oliveira R. C. , Oliveira R. C. , Janson G. , and Valarelli F. P. , Comparison of profile attractiveness between class III orthodontic camouflage and predictive tracing of Orthognathic surgery, International Journal of Dentistry. (2020) 2020, no. 1, 9, 7083940, 10.1155/2020/7083940. 32963533 PMC7492899

[30] Heldt L. , Haffke E. A. , and Davis L. F. , The psychological and social aspects of orthognathic treatment, American Journal of Orthodontics. (1982) 82, no. 4, 318–328, 10.1016/0002-9416(82)90466-3, 2-s2.0-0020198696. 6961804

[31] Barreto F. A. M. and Santos J. R. R. D. C. , Virtual orthodontic setup in orthodontic camouflage planning for skeletal Class III malocclusion, Dental Press Journal of Orthodontics. (2018) 23, no. 2, 75–86, 10.1590/2177-6709.23.2.075-086.bbo, 2-s2.0-85048590656, 29898161. PMC6018445 29898161

[32] Lin J. and Gu Y. , Preliminary investigation of nonsurgical treatment of severe skeletal Class III malocclusion in the permanent dentition, Angle Orthodontist. (2003) 73, no. 4, 401–410, 12940561. 12940561 10.1043/0003-3219(2003)073<0401:PIONTO>2.0.CO;2

[33] Mulie R. M. and Hoeve A. T. , The limitations of tooth movement within the symphysis, studied with laminagraphy and standardized occlusal films, Journal of Clinical Orthodontics. (1976) 10, no. 12, 882–893, 1074868. 1074868

[34] Gininda D. M. and Khan M. I. , A radiographic analysis of mandibular symphysis dimension in black South African adult patients with differing skeletal patterns, South African Dental Journal. (2022) 77, no. 4, 208–215, 10.17159/2519-0105/2022/v77no4a3.

[35] Garib D. G. , Yatabe M. S. , Ozawa T. O. , and Silva-Filho O. G. , Morfologia alveolar sob a perspectiva da tomografia computadorizada: definindo os limites biológicos para a movimentação dentária, Dental Press Journal of Orthodontics. (2010) 15, no. 5, 192–205, 10.1590/S2176-94512010000500023, 2-s2.0-84855521894.

[36] Jacobson A. , The “Wits” appraisal of jaw disharmony, American Journal of Orthodontics. (1975) 67, no. 2, 125–138, 10.1016/0002-9416(75)90065-2, 2-s2.0-0016467507, 1054214. 1054214

[37] Hussels W. and Nanda R. S. , Analysis of factors affecting angle ANB, American Journal of Orthodontics and Dentofacial Orthopedics. (1984) 85, no. 5, 411–423, 10.1016/0002-9416(84)90162-3, 2-s2.0-0021433574. 6586080

[38] de Alba Y. , Levy J. A. , Caputo A. A. , and Chaconas S. J. , Effects of orthodontic intermaxillary Class III mechanics on craniofacial structures. Part I–photoerlastic analysis, Angle Orthodontist. (1979) 49, no. 1, 10.1043/0003-3219(1979)049<0021:eooici>2.0.co;2. 283706

[39] de Alba Y. , Levy J. A. , Caputo A. A. , and Chaconas S. J. , Effects of orthodontic intermaxillary class III mechanics on craniofacial structures. Part II–computerized cephalometrics, Angle Orthodontist. (1979) 49, no. 1, 10.1043/0003-3219(1979)049<0029:eooici>2.0.co;2. 283707

[40] Nanda R. , Dr. Ravindra Nanda on his treatment philosophy Part - I, Journal of Indian Orthodontic Society. (2005) 39, no. 2, 68–78, 10.1177/0974909820050202.

[41] Sameshima G. T. and Asgarifar K. O. , Assessment of root resorption and root shape: periapical vs panoramic films, Angle Orthodontist. (2001) 71, no. 3, 185–189, 11407770. 11407770 10.1043/0003-3219(2001)071<0185:AORRAR>2.0.CO;2

